# Co-Culture of Human Bronchial Fibroblasts and CD4+ T Cells Increases Th17 Cytokine Signature

**DOI:** 10.1371/journal.pone.0081983

**Published:** 2013-12-05

**Authors:** Lionel Loubaki, Ikhlass Hadj-Salem, Raouia Fakhfakh, Eric Jacques, Sophie Plante, Marc Boisvert, Fawzi Aoudjit, Jamila Chakir

**Affiliations:** 1 Centre de recherche, Institut Universitaire de Cardiologie et de Pneumologie de Québec, Université Laval, Québec, Canada; 2 Centre de recherche en rhumatologie et Immunologie, Université Laval, Québec, Canada; University of Tennessee Health Science Center, United States of America

## Abstract

**Background:**

Airway inflammation is an important characteristic of asthma and has been associated with airway remodelling and bronchial hyperreactivity. The mucosal microenvironment composed of structural cells and highly specialised extracellular matrix is able to amplify and promote inflammation. This microenvironment leads to the development and maintenance of a specific adaptive response characterized by Th2 and Th17. Bronchial fibroblasts produce multiple mediators that may play a role in maintaining and amplifying this response in asthma.

**Objective:**

To investigate the role of bronchial fibroblasts obtained from asthmatic subjects and healthy controls in regulating Th17 response by creating a local micro-environment that promotes this response in the airways.

**Methods:**

Human bronchial fibroblasts and CD4^+^T cells were isolated from atopic asthmatics and non-atopic healthy controls. CD4^+^T were co-cultured with bronchial fibroblasts of asthmatic subjects and healthy controls. RORc gene expression was detected by qPCR. Phosphorylated STAT-3 and RORγt were evaluated by western blots. Th17 phenotype was measured by flow cytometry. IL-22, IL17, IL-6 TGF-β and IL1-β were assessed by qPCR and ELISA.

**Results:**

Co-culture of CD4^+^T cells with bronchial fibroblasts significantly stimulated RORc expression and induced a significant increase in Th17 cells as characterized by the percentage of IL-17^+^/CCR6^+^ staining in asthmatic conditions. IL-17 and IL-22 were increased in both normal and asthmatic conditions with a significantly higher amount in asthmatics compared to controls. IL-6, IL-1β, TGF-β and IL-23 were significantly elevated in fibroblasts from asthmatic subjects upon co-culture with CD4^+^T cells. IL-23 stimulates IL-6 and IL-1β expression by bronchial fibroblasts.

**Conclusion:**

Interaction between bronchial fibroblasts and T cells seems to promote specifically Th17 cells profile in asthma. These results suggest that cellular interaction particularly between T cells and fibroblasts may play a pivotal role in the regulation of the inflammatory response in asthma.

## Introduction

Th17 cells are distinct population of CD4^+^ T cells that produce IL-17A, IL-17F and IL-22 [1,2] and are involved in the pathogenesis of a variety of inflammatory conditions including experimental autoimmune encephalomyelitis, collagen-induced arthritis, psoriasis, allergy and asthma [3-8]. Recent studies using murine asthma models have shown that IL-17 receptor–deficient (IL-17R-/-) mice exhibit reduced recruitment not only of neutrophils but also of eosinophils into the airways [9]. Moreover, IL-17F deficient mice showed an impaired neutrophilic response to allergen [10]. It has also been demonstrated that IL-17A-/- mice exhibit reduced Th2 responses to antigen sensitization [11].

Asthma is characterized by airway inflammation and remodelling. The mucosal microenvironment composed of structural cells and highly specialised extracellular matrix (ECM) is able to amplify and promote inflammation in the airways. *In vitro* and animal studies showed that interactions of inflammatory cells with structural cells play an important role in airway inflammation and remodelling [12,13]. 

In humans, physiological generation of Th17 in vivo is not fully understood. It seems likely that the local microenvironment of stromal cells creates an optimal cytokine milieu and provides cell-cell contact mechanisms that facilitate the generation of Th17 cells. Differentiation of Th17 in vivo requires further stimuli produced locally by stromal cells such as fibroblasts and macrophages. Thus, in arthritis, synovial macrophages support Th17 cell differentiation via a network of cytokines [14]. In skin, fibroblasts support the expansion of Th17 via IL-23 [15]. Moreover, fibroblasts can release cytokines that are involved in Th17 differentiation such as IL-6 and TGF-β [16,17]. We showed that structural cells cultured in an engineered mucosa model increase T cell survival through production of cytokines such as TGF-β [18]. IL-17, the main cytokine of Th17 is well known as a modulator of fibroblast and epithelial cell function. We have shown that IL-17 stimulated the production of different cytokines and chemokines by bronchial fibroblasts [19]. A recent study showed that co-culture of fibroblasts with T cells increased IL-17 and CD40L expression by T cells. This increase in IL-17 and CD40L stimulated the production of a specific chemokine that promotes migration of CD4^+^ memory cells [20]. We showed that interaction of T cells with bronchial fibroblasts increase production of IL-6 by bronchial fibroblasts [21]. Interaction of Th17 with bronchial fibroblasts in asthma may induce a specific cytokine profile by bronchial fibroblasts and in turn may amplify specific Th17 cytokines such as IL-17 and IL-22. Although an important body of knowledge exits about regulation of Th17, very little is known about the interplay between Th17 and the local microenvironment in asthma. This local microenvironment may restrict the production of cytokine such as IL-17 to the site of infection and may limit systemic inflammation.

In this study, we investigated the role of bronchial fibroblasts obtained from asthmatic subjects and healthy controls in regulating Th17 response by creating a local micro-environment that promotes this response in the airways.

## Methods

### Subjects

Asthmatic subjects fulfilling the American Thoracic Society criteria for asthma [22] were recruited from the Asthma Clinic at the Institut Universitaire de Pneumologie et de Cardiologie de Québec. All asthmatic subjects (mean age, 39.4 ±12.8 years; mean FEV_1_, 100 ± 0.14 %; mean PC_20_, 3.5 ± 2.7 mg/mL) were atopic with at least one positive response to common allergens on allergy skin prick tests. They used only an inhaled β_2-_agonist on demand and did not use inhaled or systemic corticosteroids. In the month preceding the study, none of the subjects reported a respiratory infection or an increase in asthma symptoms. Healthy subjects (mean age, 40.1 ± 11years; mean FEV_1_, 107 ± 0.15 %; mean PC_20_, 83.8 ± 16.1 mg/mL) were non-atopic non-smokers with no history of asthma or atopy. The study was approved by the Institut Universitaire de Cardiologie et de Pneumologie (Québec, Canada) ethics committee and all subjects signed an informed consent form.

### Bronchial fibroblast isolation and culture

Fibroblasts were isolated from bronchial biopsies as described previously [23]. Cultured fibroblasts were identified and characterized by immunofluorescence and flow cytometry using anti-vimentin and a mouse anti-human fibroblast antigen Ab-1 antibody (Calbiochem, San Diego, CA, USA). The purity of fibroblast cell culture was 98% [24]. Cells were grown in Dulbecco's modified Eagle’s medium (DMEM) supplemented with 100 units/ml penicillin/streptomycin (Gibco, Burlington, ON, Canada) and 10% FBS (Gibco). The media was changed three times a week. Cells were used at early passages to keep their phenotype [25].

### CD4^+^ T lymphocyte isolation

Healthy and asthmatic subjects, from whom structural bronchial fibroblast cells were derived, donated blood which was used as a source of CD4^+^ T lymphocytes. Mononuclear cells were isolated from peripheral blood using Ficoll-Paque density gradient (GE Healthcare, Buckinghamshire, UK) by centrifugation at 700g for 20 minutes (min). Cells were washed with RPMI containing 10% of heat-inactivated FBS culture medium and incubated in a culture flask to allow monocytes to adhere. Non-adhering cell suspensions were then collected and CD4^+^ T lymphocytes were purified using CD4+ T Cell isolation kit II (Miltenyi Biotec, Bergisch Gladbach, Germany). To assess the purity, cells were stained with anti-CD4-FITC coupled antibody (BD Bioscience, San Diego, CA, USA) and analyzed by flow cytometry that revealed 97% of the purified cells were CD4^+^ T cells.

### Fibroblasts and CD4^+^ T lymphocyte co-cultures

Bronchial fibroblasts from healthy (HBF) and asthmatic subjects (ABF) were plated at 2x10^5^ cells per well in a 6-well culture plates in DMEM medium and allowed to adhere overnight at 37°C in a humidified incubator and 5% CO_2_ atmosphere. The following day, 2x10^6^ CD4^+^ T cells were added to bronchial fibroblasts in transwell system. T cells were harvested and fibroblasts were then detached by trypsin and the presence of contaminating T cells was evaluated by flow cytometry using anti-CD4. The purity of the retrieved fibroblasts was 99%. T cells were then stimulated with anti-CD3/CD28 dynabeads (Life technologies, Burlington, ON.) for 24 hours and culture supernatants were collected.

### Real Time PCR analysis

IL-17, IL-22, IL-6, IL-1-β, and GAPDH were amplified by quantitative real time PCR using the SybrGreen reagent (Qiagen) on an Opticon-2 system (MJ Research Inc, Waltham, MA, USA) as we previously described [26]. All primers forward and reverse sequences are listed in Table 1. Normalized expressions were calculated by using the calculated efficiency of each PCR reaction with a cDNA standard curve. 

**Table 1:** Primers used for quantitative real time PCR.

CCL-20 fw	TTT GCT CCT GGC TGC TTT GATGT
CCL-20 rv	GTT TTG GAT TTG CGC ACA CAG AC
Foxp3 fw	CTACGCCACGCTCATCCGCTGG
Foxp3 rv	GTAGGGTTGGAACACCTGCTGGG
GAPDH fw	ATG CAA CGG ATT TGG TCG TAT
GAPDH Rrv	CTG AGG GCT GAG ATG CCG
GATA-3 fw	ACC GGC TTC GGA TGCAA
GATA-3 rv	TGC TCT CCT GGC TGC AGAC
IL-1β fw	GCA ATG AGG ATG ACT TGT TCT TTG
IL-1β rv	AGG TCC AGG TCC TGG AA
IL-6 fw	TCT CCA CAA GCG CCT TCG
IL-6 rv	CTG AGG GCT GAG ATG CCG
IL-17 fw	ATCTCCACCGCAATGAGGAC
IL-17rv	GTG GAC AAT CGG GGT GACAC
IL-21 fw	TGT GAATGACTTGGTCCCTGAA
IL-21 rv	AACAGGAAAAAGCTGACCACTCA
IL-22 fw	TGAATAACTAACCCCCTTTTCCCTG
IL-22 rv	TGGCTTCCCATCTTCCTT TTG
IL-23p19 fw	GAGCCTTCTCTGCTCCCTCCTGAT
IL-23p19 rv	AGTTGGCTGAGGCCCAGTAG
RORc fw	TTTTCCGAGGATGAGATTGC
RORc rv	CTTTCCACATGCTGGCTACA
T-bet fw	AACACAGGAGCGCACTGGAT
T-bet rv	TGCTCTCCTGGCTGCAGAC

### Cytokine measurements

IL-7A, IL-22, IL-6, TGF- β and IL-23 were measured in culture supernatants by using Quantikine HS Human ELISA Kits following the manufacturer’s instructions. (R&D Systems, Inc, Minneapolis, MN).

### Flow cytometry analysis

After the co-culture, T cells were collected by extensive washing, centrifuged at 700g for 10 min and then incubated for 4h with a golgistop containing Monensin (BD), at 37°C in a humidified incubator and 5% CO_2_ atmosphere, prior to the staining. Following the incubation, T cells were recovered and centrifuged once more at 700 g for 10 min before being stained with 10 μg/ml monoclonal mouse anti-human antibodies specific for anti-CD4 (BD), anti-IL-17-Pecrp and anti-CCR6-PE (R&D Systems, Mineapolis, MN). 

### Western Blot

Lymphocytes were collected before or after 24 hours co-culture with bronchial fibroblasts and lysed in lysis buffer (25mM Tris-HCl PH 8.0, 150 mM NaCl, 1mM EDTA, 0.1% SDS, 0.05% sodium deoxycholate, 1% Triton X-100, 3 mM PMSF) for one hour at 4°C. Protein-containing supernatants were collected and protein concentration was determined using the Bradford assay. Proteins (20 μg) in reducing sample buffer (containing 5% of 2-mercaptoethanol) were migrated onto 10% SDS-PAGE gels and transferred to PVDF membranes. Antibodies anti-RORγt (Igenex, San-Diego, CA, USA; 1:500); anti-phosporylated STAT-3 (Cell Signaling Technology, Danvers, MA, USA; 1:5000), anti-STAT3 (BD; 1:5000) or anti-β-actin (Sigma-aldricht laboratories,Oakville, ON, Canada; 1:10 000) were blotted overnight and revealed with appropriate peroxydase-conjugated secondary antibodies for one hour at room temperature. Luminescence was visualized with autoradiography on X-Omat blue films. Semi-quantitavive analyses were performed by measuring the relative intensity of each band, subtracting the background level of an empty blue film and calculating ratios of RORγt on β-actin and ratios of phosphorylated STAT3 on total STAT3 for each condition and each set of experiment.

### Statistical analysis

All experiments were performed three times. We used SAS software package and a statistician performed the analysis. *A posteriori*, multiple comparisons were performed using the Tukey-Kramer test. Mean values ± SEM of quantitative variables were used as representative measures. All reported *p* values were two-sided and were declared significant at 0.05 levels.

## Results

### Effect of co-culture on the expression of CD4^+^T cell lineage specific transcription factors

**Figure 1:**
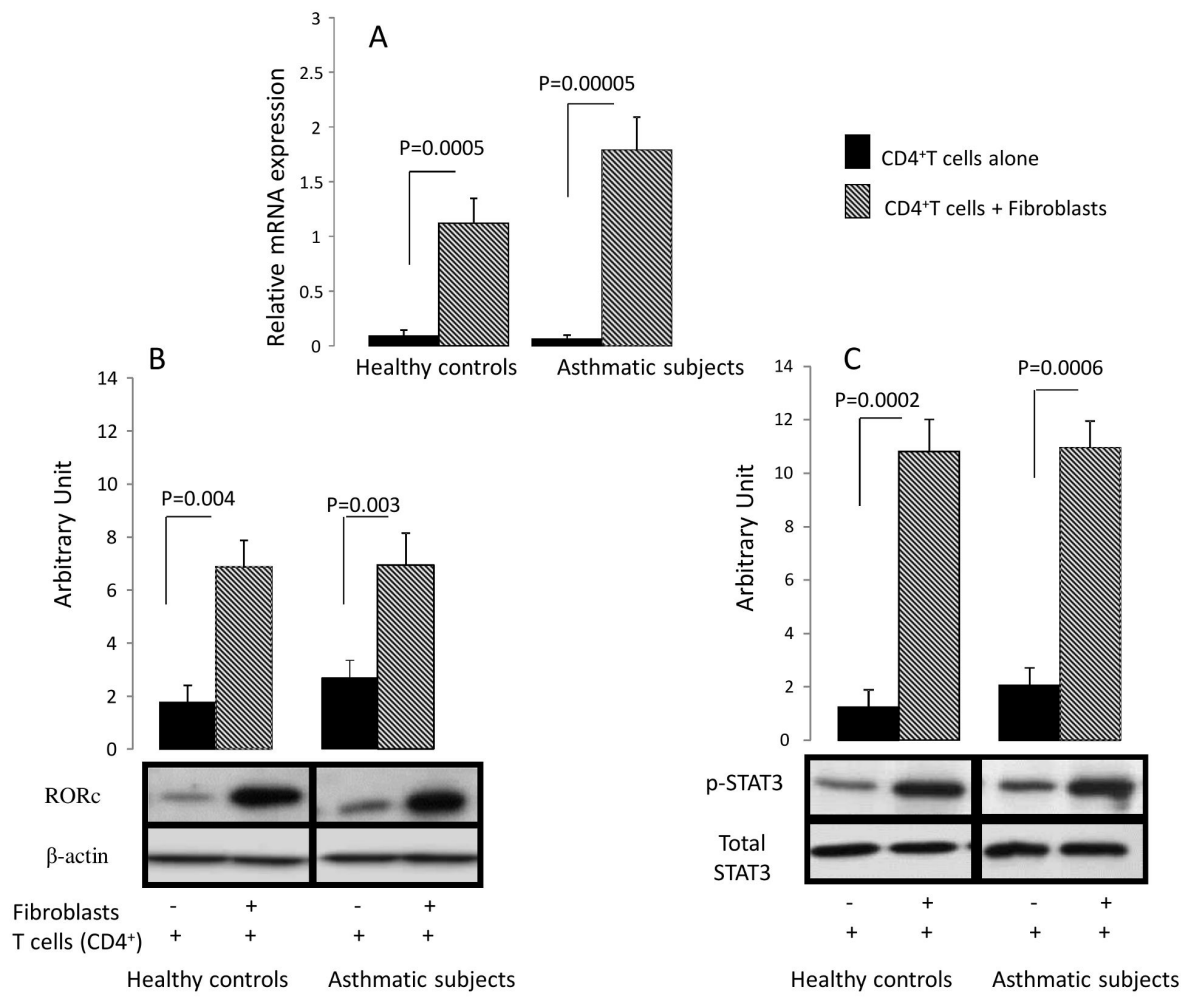
Effect of human bronchial fibroblasts on CD4^+^ T cells transcription factor expression. (*A*) Quantitative PCR analysis of RORc mRNA expression of CD4^+^ T cells. Fibroblasts and CD4^+^ T cells were cocultured during 14 hours, and then T cells were removed by PBS washing. RNA was then extracted from CD4^+^T cells. Data are presented as mean ± SEM of 8 asthmatics and 8 healthy controls. (*B*,*C*) RORγt and STAT3 expression in CD4^+^ T cells following coculture. Results are expressed as densitometric intensity of RORγt product over the densitometric intensity of the β-actin product or densitometric intensity of pSTAT3 over that of the total STAT3. Data are presented as mean ± SEM of 4 experiments.

T-bet, GATA-3, Foxp3 and RORc gene expression by T cells was measured following co-culture with bronchial fibroblasts. T-bet, GATA-3 and Foxp3 mRNA expression were not modulated by the co-culture (data not shown). RORc mRNA significantly increased in T cells after co-culture with both bronchial fibroblasts from asthmatic (0.06 ± 0.04 for T cells alone to 1.79 ± 0.30 mRNA relative expression when cocultured with asthmatic fibroblasts; p=0.00005) and healthy subjects (0.06 ± 0.05 for T cells alone to 1.12 ± 0.22 mRNA relative expression when co-cultured with healthy fibroblasts; p=0.0005). The effect was more pronounced in asthmatics compared to controls (Figure 1-A). In addition, RORγt and phosphorylated STAT3 expression were increased at protein level in both asthmatic and healthy controls’ CD4^+^ T cells following co-culture (Figure 1- B and C). 

**Figure 2:**
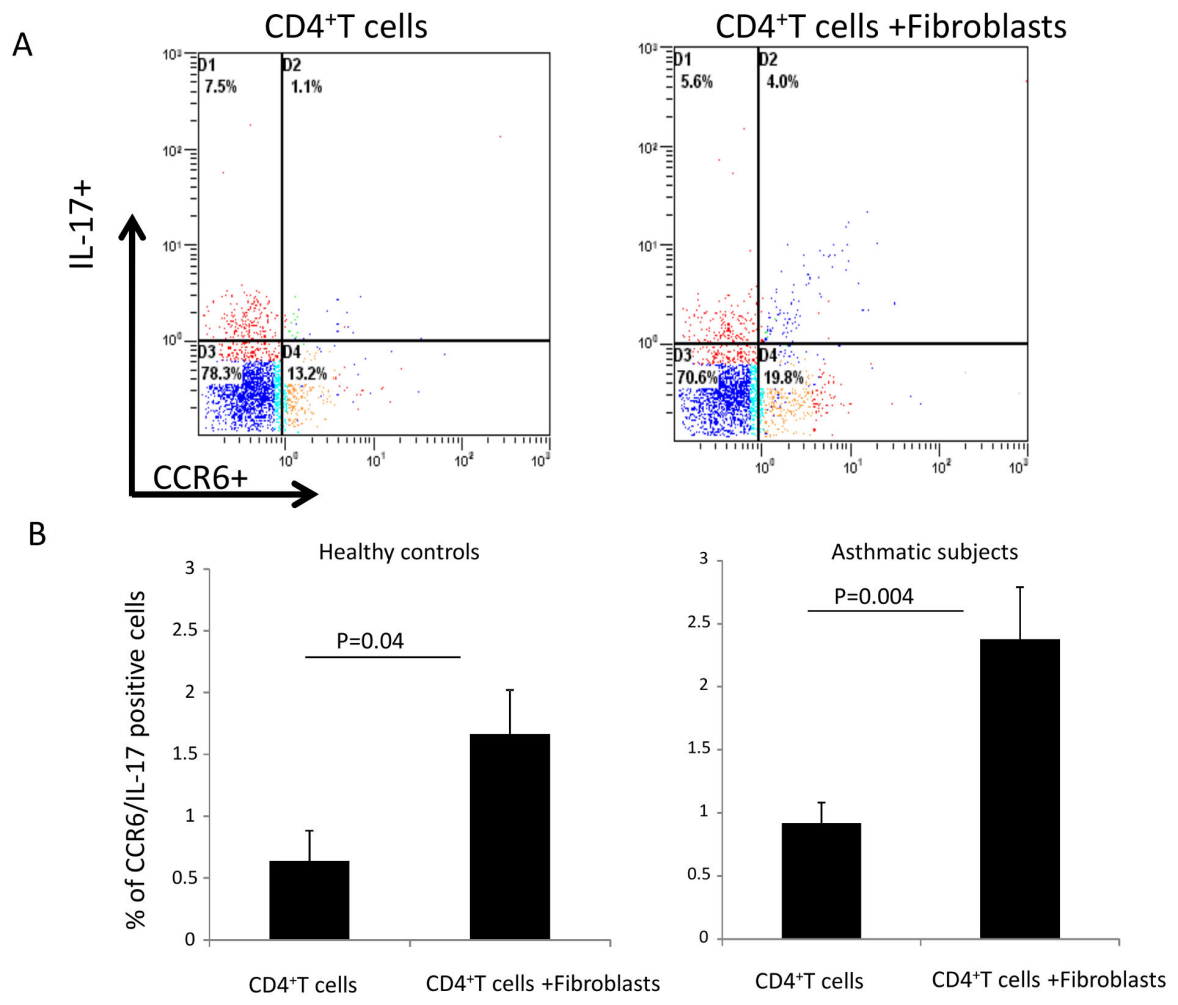
Effect of the co-culture on Th17 cells phenotype (IL17^+^/CCR6^+^). (*A*, *B*) Bronchial fibroblasts and CD4^+^ T cells were co-cultured during 4 days and then T cells were retrieved by extensive PBS washing. CD4^+^ T cells were submitted to a flow cytometry analysis using both IL-17 and CCR6 monoclonal antibody. Results are showed as percentage of positive cells for both IL-17 and CCR6 (IL-17^+^/CCR6^+^). Data are representative of 8 asthmatics and 8 healthy controls.

Flow cytometry analysis shows that co-culture of CD4^+^ T cells with fibroblasts induced a significant increase in Th17 cells as characterized by the percentage of IL-17+/CCR6+ staining at day 4 (T cells from healthy controls alone 0.64 ± 0.22 % to 1.67 ± 0.37 % of IL-17+/CCR6+ T cells when co-cultured with healthy fibroblasts versus 0.92 ± 0.16% for T cells from asthmatic subjects to 2.38 ± 0.41% of IL-17+/CCR6+ T cells when co-cultured with asthmatic fibroblasts). The effect is more important in asthmatic conditions (Figure 2 A and B). 

### Effect of co-culture on Th17 lineage associated cytokines signature

**Figure 3:**
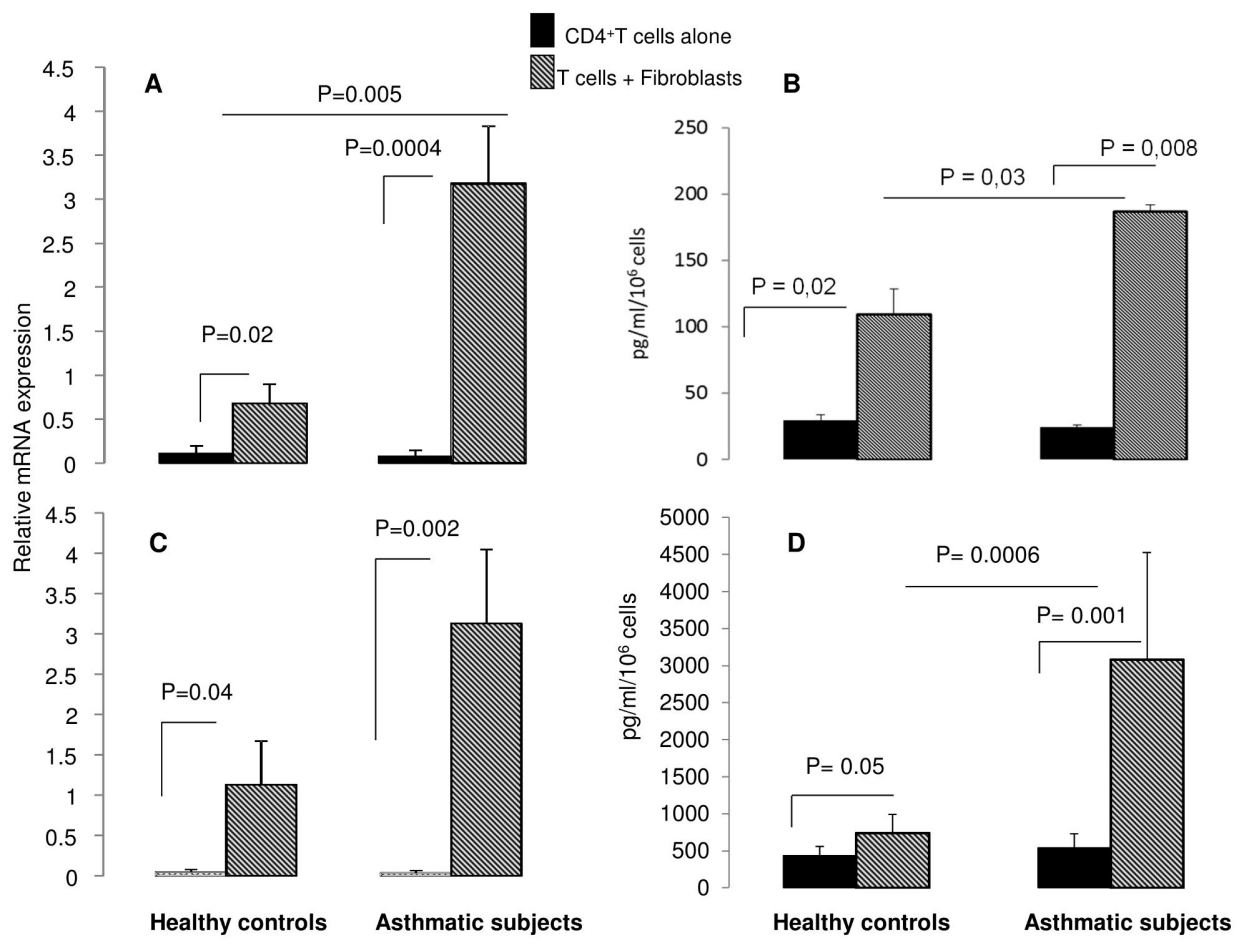
Effect of bronchial fibroblasts on Th17 cell lineage associated cytokines. Quantitative PCR analysis of IL-17 and IL-22 gene expression by CD4^+^ T cells following co-culture with fibroblasts (*A* and *B*). C and D represent IL-17 and IL-22 protein levels in co-culture supernatants Data are presented as mean ± SEM of 8 experiments.

Figure 3-A shows that IL-17 gene expression is increased when CD4^+^ T cells were co-cultured with both healthy (from 0.11 ± 0.08 for CD4^+^ T cells alone to 0.68 ± 021 mRNA relative expression when co-cultured with healthy fibroblasts; p=0.02) and asthmatic CD4^+^ T cells (from 0.07 ± 0.06 for CD4+ T cells alone to 3.18 ± 0.65 mRNA relative expression when co-cultured with asthmatic fibroblasts; p=0.0004; Figure 3- A). This increase is significantly higher (p = 0,005) when T cells were co-cultured with fibroblasts from asthmatic than from controls. Figure 3-B shows that IL-17 protein release in co-culture supernatants was increased in both normal and asthmatic conditions with a significantly higher amount in asthmatics compared to controls (p = 0.03)

Figure 3-C and D shows IL-22 gene and protein expression. IL-22 expression is increased in T cells co-cultured with fibroblasts from healthy controls (0.04 ± 0.03 to 1.13 ± 0.5 mRNA relative expression; p=0.04). In asthmatics, this increase is significantly higher (0.04 ± 0.02 to 3.13 ± 0.85 mRNA relative, p = 0,002) than in healthy controls. At protein level, IL-22 release is significantly higher when T cells were co-cultured with fibroblasts from asthmatic compared to healthy controls (p = 0.0006).

### Effect of co-culture on Th17 differentiating cytokines expression

**Figure 4:**
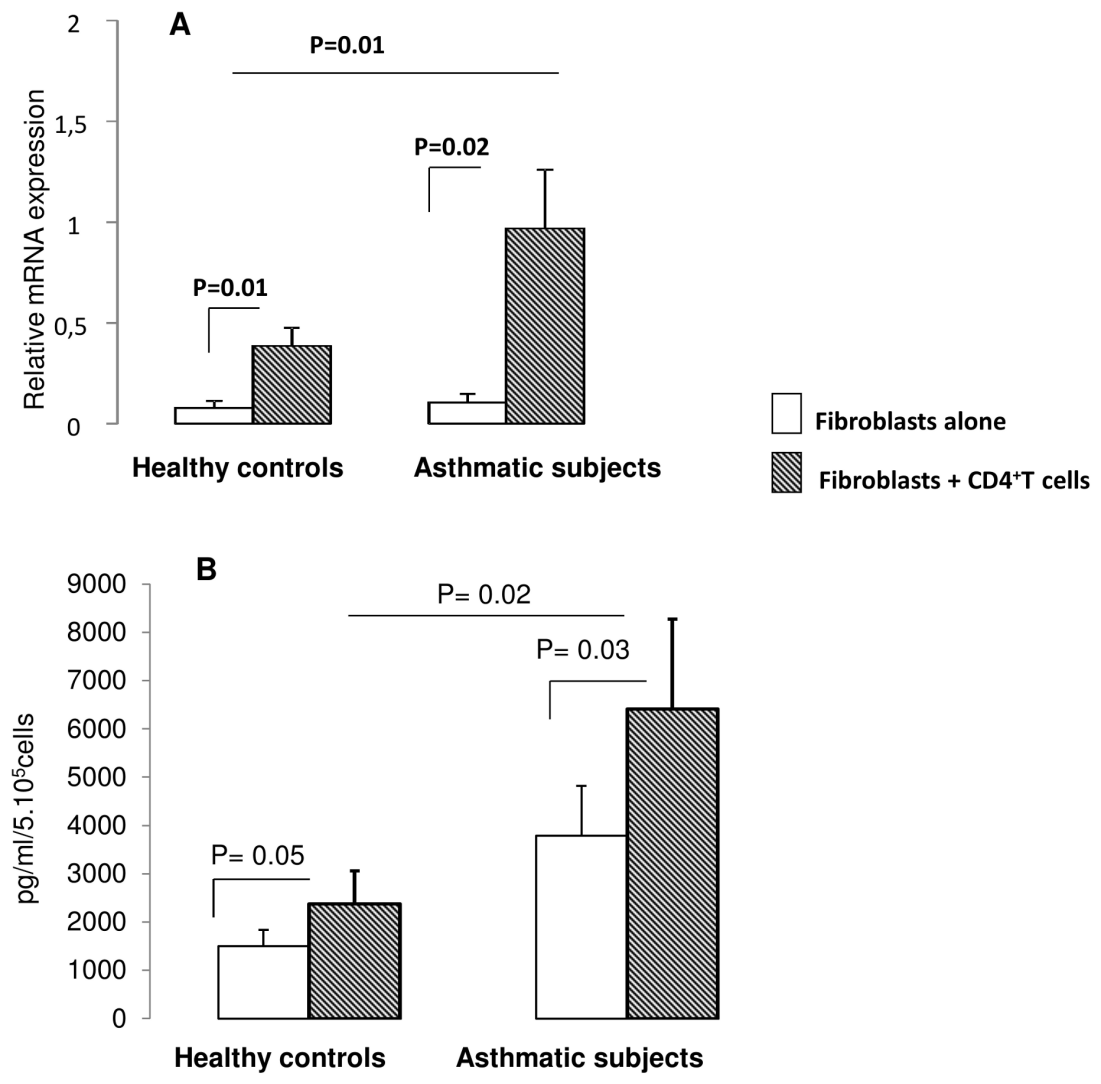
Effect of CD4^+^ T cells on IL-6 expression by fibroblasts following co-culture. Quantitative PCR analysis of IL-6 gene expression by bronchial fibroblasts following co-culture with CD4^+^ T cells (*A*). B- represent IL-6 protein levels in co-culture supernatants Data are presented as mean ± SEM of 8 experiments.

**Figure 5:**
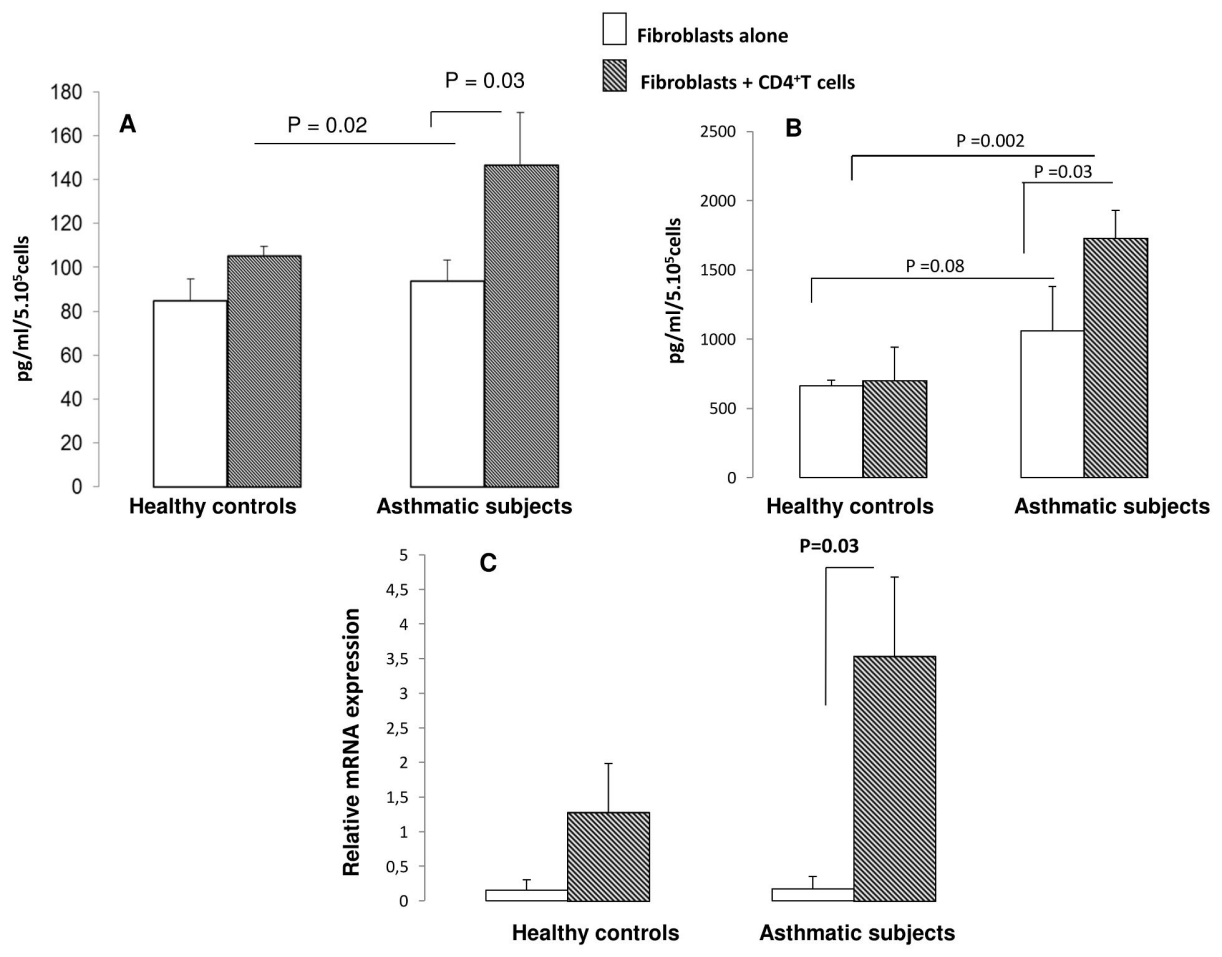
IL-23, TGF-β and IL-1β expression by fibroblasts following co-culture with CD4^+^ T cells. (*A*, *B*) IL-23 and TGF-β production were measured in culture supernatants after 24h of coculture. Data are presented as mean ± SEM of 6 experiments. (*C*) Quantitative PCR analysis of IL-1β mRNA expression by bronchial fibroblasts co-cultured with CD4^+^ T cells. Data are presented as mean ± SEM of 6 experiments.

Co-culture of CD4^+^ T cells with fibroblasts induced a significant increase in IL-6 gene expression by fibroblasts from controls (p= 0.01) and asthmatics (p= 0.02 Figure 4 A). The effect is significantly higher in fibroblasts from compared to healthy controls. Figure 5-B shows IL-6 protein release in co-culture. In asthmatic condition, the amount of IL-6 is significantly higher than in control conditions (p =0.02). 

IL-23 production by bronchial fibroblasts was significantly increased in co-culture supernatants of asthmatics (94 ± 4 to 147 ± 23 pg/ml, p = 0.03) whereas no significant increase was shown in control conditions (85 ±10to 105 ±9 pg/ml) (Figure 5-A). Moreover, we found that TGF-β production was significantly higher in co-culture supernatants of asthmatics (1059 ± 321 to 1728 ± 200 pg/ml, p = 0.03). No change was observed in control conditions (Figure 5-B).

Figure 5-C shows that gene expression of IL-1β by fibroblasts from healthy controls was not affected by the co-culture (from 0.15 ± 0.15 for fibroblasts alone to 1.28 ± 0.69 mRNA relative expression when co-cultured with healthy T cells; p=0.15). Whereas, IL-1β expression was significantly increased in fibroblasts from asthmatics following coculture with CD4^+^ T cells (from 0.17 ± 0.17 for fibroblasts alone to 3.53 ± 1.13 mRNA relative expression when co-cultured with asthmatic T cells; p=0.03). 

**Figure 6:**
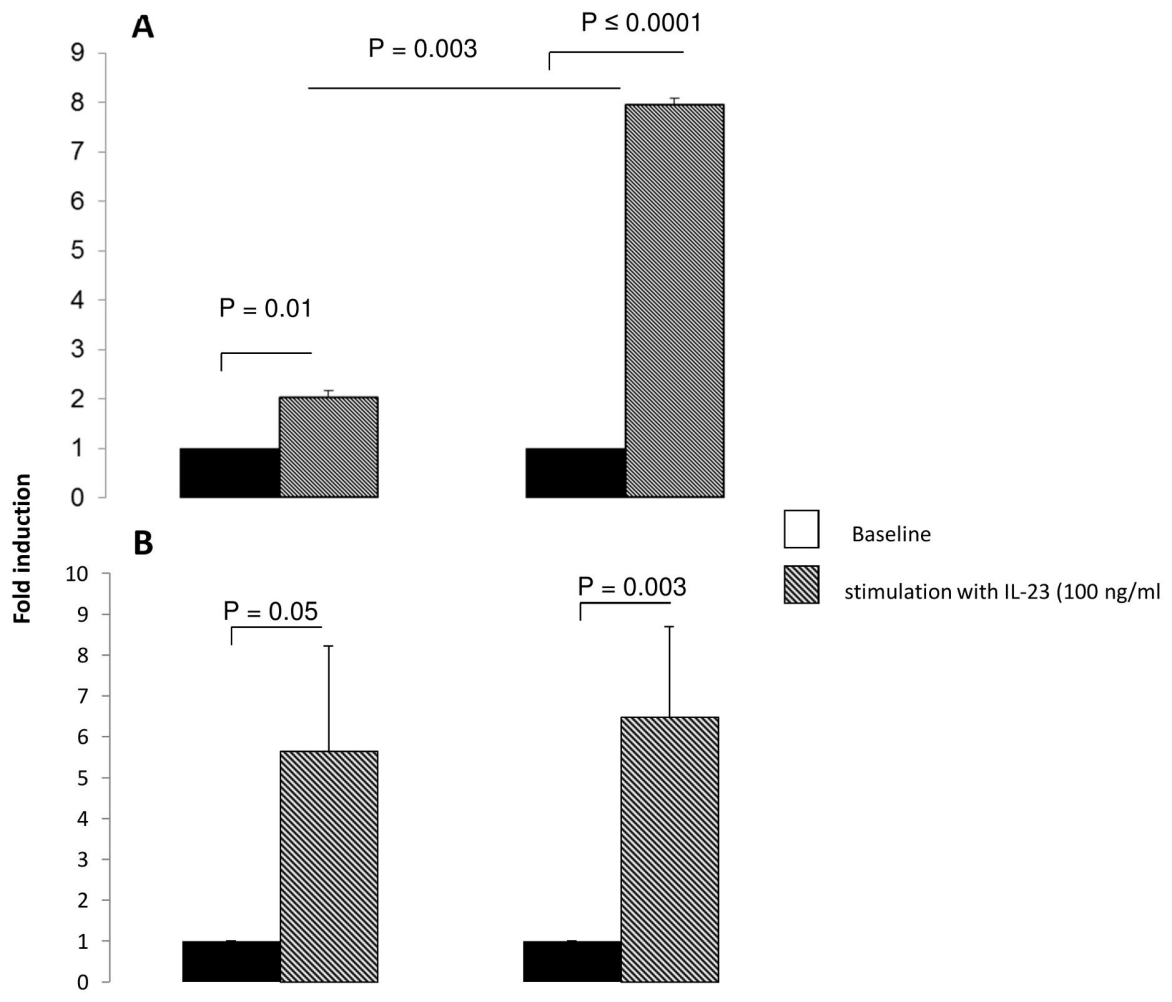
IL-23 stimulation of bronchial fibroblasts. Quantitative PCR analysis of IL-6 (*A*) and IL-1β (*B*) mRNA expression by bronchial fibroblasts following stimulation with 100 ng/mL of recombinant IL-23. Cells were stimulated for 6 hours and total RNAs were extracted and qRT-PCRs were performed. Data are presented as mean ± SEM of 6 experiments.

**Figure 7:**
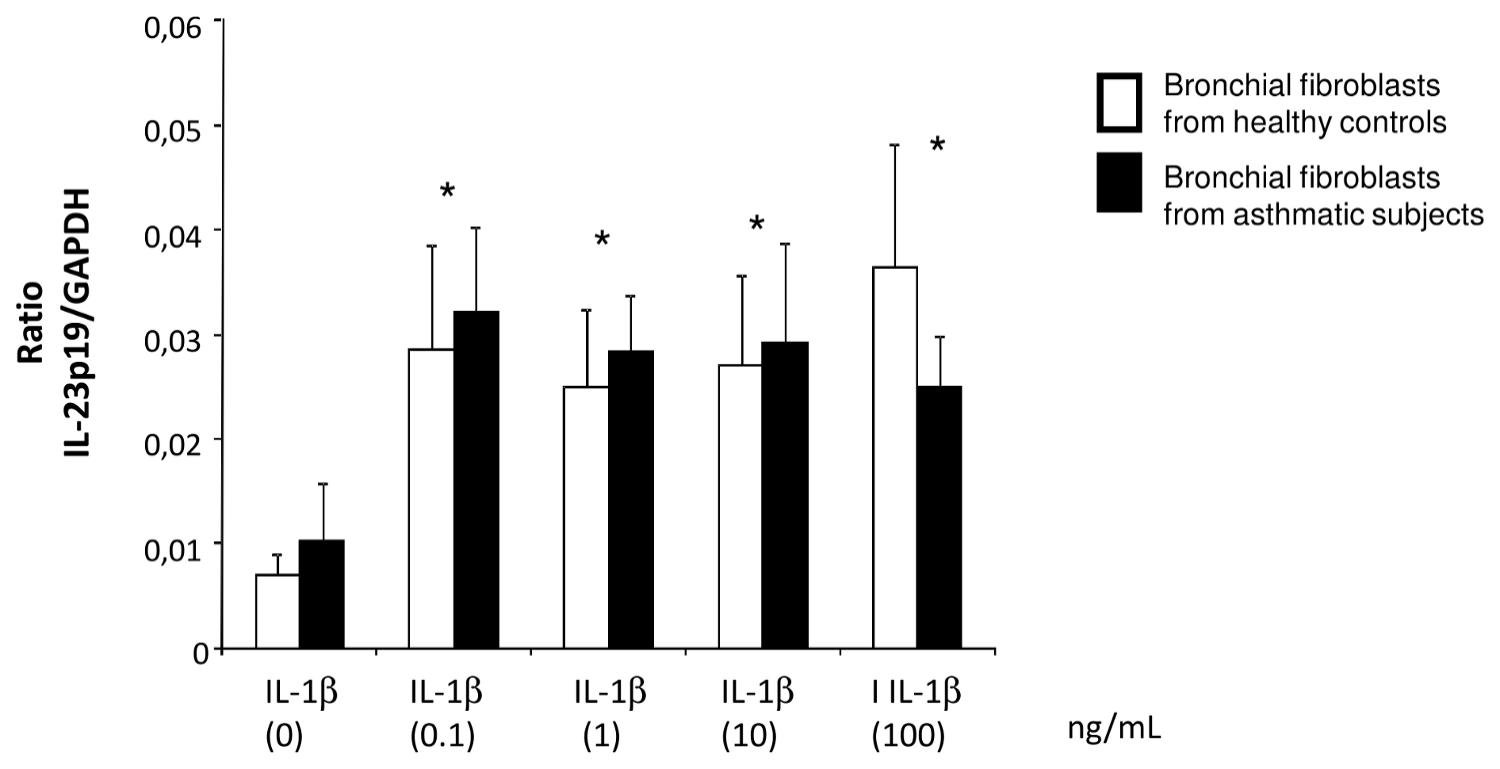
IL-1β stimulates bronchial fibroblasts expression of IL-23p19 mRNA. Quantitative PCR analysis of IL-23p19 mRNA expression in bronchial fibroblasts stimulated with different doses of IL-1β. Data are presented as mean ± SEM of 8 experiments. * p< 0.001.

To identify potential mechanisms involved in the preservation of Th17 cells induced by the co-culture IL-23 receptor (IL-23R) expression was assessed by flow cytometry. We found that both fibroblasts from healthy controls (48.3 ± 6.18 % of IL-23R+ cells) and asthmatics (55.10 ± 6.26 % of IL-23R+ cells) express IL-23R at their surface membrane (data not shown). In addition, no significant difference was found in term of mean fluorescence intensity between the two populations. Stimulation of bronchial fibroblasts with a recombinant IL-23 (rIL-23) at 100 ng/ml significantly increased IL-6 and IL-1β gene expression by fibroblasts of asthmatic subjects (Figure 6-A and B), whereas, TGF-β expression was not affected (data not shown) suggesting that an auto regulation loop involving IL-23/IL-1β axis may promote a persistent Th17 cell phenotype in our system. Thus, stimulation of bronchial fibroblasts with increasing concentrations of IL-1β induced a significant increase in IL23 gene expression in fibroblasts from both asthmatic and healthy subjects (Figure 7).

## Discussion

In this study, we showed that CD4^+^ T cells and bronchial fibroblasts interaction promotes the production of cytokines involved in Th17 immune response. This effect was significantly pronounced in the asthmatic condition. Bronchial fibroblasts from asthmatic subjects create a local microenvironment characterized by an increased expression of IL-6, IL-1β, TGF-β and IL-23 which are known as cytokines promoting the persistence and differentiation of Th17 cells. 

The response to tissue damage involves a carefully choreographed series of interactions between diverse cellular, cytokines, chemokines and connective tissue elements. Inflammation normally resolves via an active process involving naturally occurring anti-inflammatory mediators which gradually replace pro inflammatory mediators. In chronic inflammatory diseases such as asthma, a failure to resolve the immune response after the clearance of the inciting agent is observed. This chronic state is characterized by several modifications of the homeostatic environment of the tissue, including abnormal cytokine production, persistent infiltration of immune cells and an increased deposition of matrix proteins produced by stromal cells such as fibroblasts and smooth muscle cells [27].. Fibroblasts play an important role in inflammation resolution phase as they attempt to repair damaged tissues. Thus, it is becoming increasingly clear that fibroblasts are not passive players in immune responses and several evidences showed that, through the release of soluble signals and/or direct interactions with other cells, they may serve as potential regulators of the local inflammatory response. Indeed, it has been reported that normal human lung fibroblasts cocultured with LPS-activated monocytes modulate their IL-10 and IL-12 production [28,29]. Furthermore, we showed that fibroblasts derived from asthmatics have the capacity to increase IL-4 production by mast cells and more recently we found that crosstalk between T cells and fibroblasts derived from both healthy and asthmatic subject’s leads to an increased IL-6 expression and production [13,26].

We found that crosstalk between CD4^+^ T cells and bronchial fibroblasts result in increased expression of the Th17 lineage specific transcription factor RORγt whereas, other transcription factors specific for Th1 (T-bet), Th2 (GATA-3) and T reg (Foxp3) T helper cells lineage were not modulated suggesting that, this interaction promote the engagement of CD4^+^ T cells into Th17 lineage pathway. These results are supported by the finding that the creation, by fibroblasts, of a local microenvironment, which was characterized by an increased expression of Th17 differentiating cytokines including IL6 and IL-1β and maintenance cytokines such as IL-23, drives an engagement into Th17 pathway. This effect was more pronounced with asthmatic co-culture conditions. It is well established that the differentiation of Th17 cells is initiated by TGF-β and IL-6 and requires IL-23 [30]. More specifically, TGF-β and IL-6 function together to induce Th17-cell differentiation, as both cytokines are necessary for the up-regulation of RORγt and IL-23R expression. Subsequently, IL-23 induces the expansion and maturation of differentiated Th17 cell populations and is required for the acquisition of a complete effector function [31,32].

RORγt and STAT-3 are key transcription factors that regulate the acquisition of Th17 lineage specific signature. RORγt and STAT3 regulate both *in vivo* and *in vitro* IL-17 expression [35-37]. Based on RORγt expression and STAT3 activation in CD4^+^ T cells following coculture, we aimed to determine if cytokines characteristic of Th17 signature immune response were also modulated by the coculture. We found that CD4^+^ T cells expression of Th17-associated cytokines IL-17 and IL-22 was significantly increased, indicating that following co-culture CD4^+^ T cells are directed to acquire the expression of Th17-phenotype. Interestingly, we found that IL-17 and IL-22 production was significantly higher in asthmatic compare to healthy CD4^+^ T cells upon co-culture. The explanation for this finding may come from asthmatic fibroblasts characteristics. Indeed, fibroblasts have been shown as an important regulator of the switch from acute to chronic inflammation and exhibit considerable diversity and topographic differentiation [38]. We and others showed that fibroblasts from asthmatic subjects retain positional memory even when cultured in vitro [39]. We showed that IL-6 production was highly produced by asthmatic compared to healthy bronchial fibroblasts following coculture, thus supporting the already described role of IL-6 in dictating the transition from acute to chronic inflammation [40]. Also, IL-1β expression was significantly increased in asthmatic fibroblasts when no response was observed from healthy bronchial fibroblasts upon coculture. These data are in accordance with previous ones that reported an association between IL-1β expression and remodelling features such as tissue fibrosis in the airways which often follow chronic inflammation [41,42].

As T cells/fibroblasts interaction seems to promote the acquisition of Th17 lineage characteristics, we performed co-culture during 4 days in order to determine whether or not this phenomenon originate from *de novo* differentiation or from the maintenance of a pre-exiting Th17 cells. We found that, both healthy and asthmatics CD4^+^ T cells had a maintain percentage of cells positive for IL-17^+^/CCR6^+^ when cocultured with fibroblasts. This result may be explained by the increased production of Th17 differentiating and maintenance factors, particularly IL-23 who plays an important role in the maintenance of Th17 phenotype [43]. This particular local microenvironment results in an increased Th17 phenotype as characterized by both IL-17 and CCR6 expression on CD4^+^ T cells. In addition, we performed a 4 days co-culture with naïve T cells (data not shown) which showed no increased percentage of cells positive for IL-17^+^/CCR6^+^ suggesting that the observed IL-17^+^/CCR6^+^ increased population at day 4 of coculture does not originate from *de novo* differentiation of naive CD4^+^ T cells but probably comes from the maintain of Th17 pre-existing population. The mechanism involved in this maintained Th17 phenotype may involve both IL-23 and IL-21. Indeed, IL-23 is well recognized as an important factor for maintain of Th17 immune response as revealed by the absence of Th17 cells in IL-23p19 and IL-23R deficient mice while, IL-21 produced by Th17 cells leads these later one to increase their own differentiation and expansion [4,33,34,43]. In addition we found that both fibroblasts from healthy and asthmatic subjects express a functional IL-23R and stimulation of fibroblasts with IL-23 leads to a significant increase of IL-6 and IL-1β expression. In the same way, we showed that IL-1β also stimulate IL-23 expression suggesting an auto-regulation loop that may contribute to the maintenance of IL-17^+^/CCR6^+^ population in coculture condition. This hypothesis fits with available data suggesting that both IL-1β and IL-23 regulate each other in order to induce Th17 differentiation, maturation and expansion [44,45]. 

**Figure 8:**
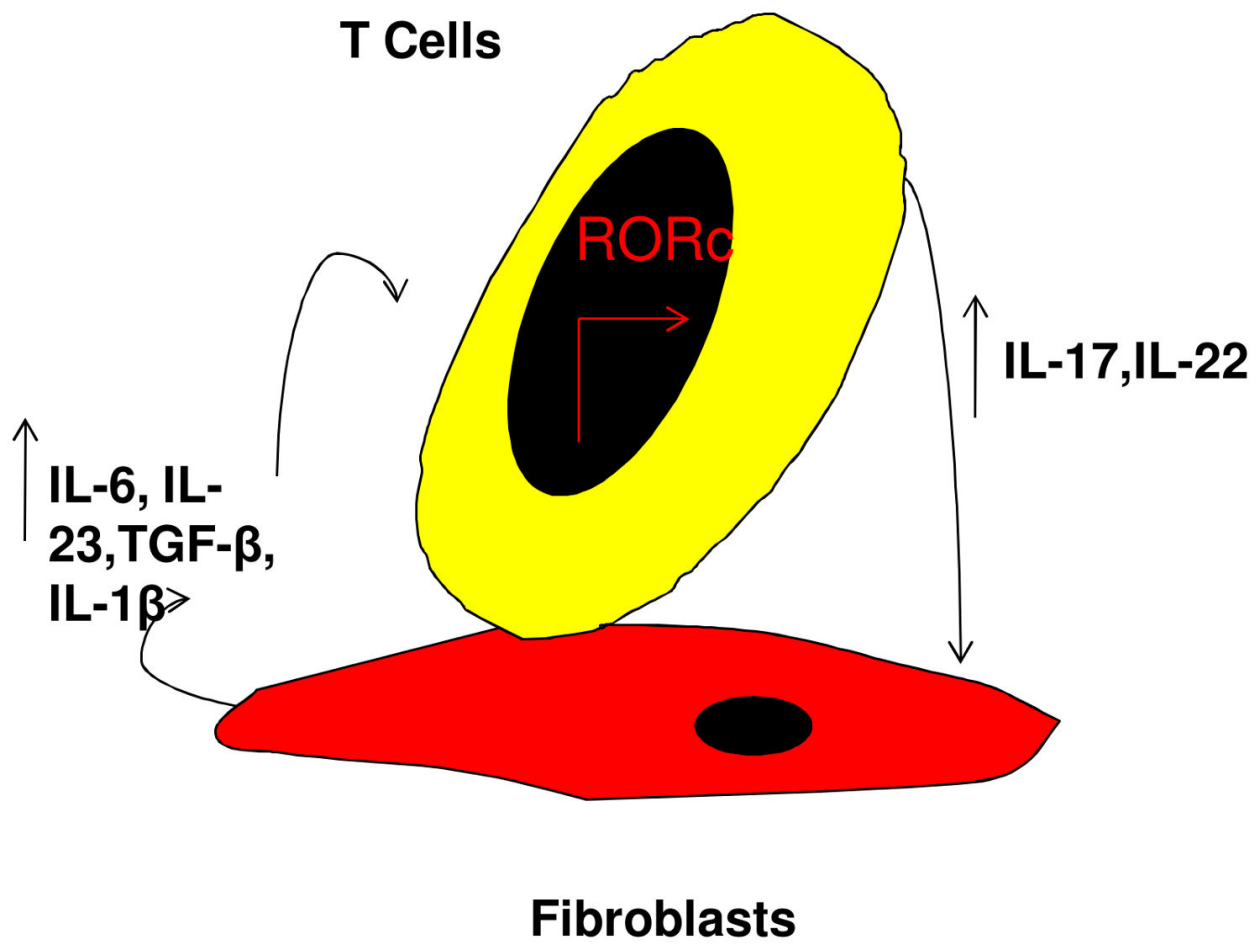
Illustration of how bronchial fibroblasts could participate in Th17 cells phenotype maintains. (1) CD4^+^ T cells stimulate fibroblasts to produce Th17 differentiating cytokines, thus creating a cytokine milieu prone to induce Th17 lineage specific transcription factor RORc and associated cytokines including IL-17 and IL-22 (2). Preservation of Th17 phenotype may involve an autoregulation loop and IL-23 by stimulation of IL-6, TGF-β and IL-1β expression (1).

In summary, our study highlighted the fact that the crosstalk between T cells and fibroblasts drives the later to create a specific local microenvironment that will promote a Th17 immune response in asthma. Figure 8 summarizes the possible crosstalk between bronchial fibroblasts and T cells in the airways.
